# T cells and ILC2s are major effector cells in influenza‐induced exacerbation of allergic airway inflammation in mice

**DOI:** 10.1002/eji.201747421

**Published:** 2018-06-11

**Authors:** Bobby W. S. Li, Marjolein J. W. de Bruijn, Melanie Lukkes, Menno van Nimwegen, Ingrid M. Bergen, Alex KleinJan, Corine H. GeurtsvanKessel, Arno Andeweg, Guus F. Rimmelzwaan, Rudi W. Hendriks

**Affiliations:** ^1^ Department of Pulmonary Medicine Erasmus MC Rotterdam Rotterdam the Netherlands; ^2^ Department of Viroscience Erasmus MC Rotterdam Rotterdam the Netherlands

**Keywords:** Allergy, Allergic airway inflammation, Exacerbation, ILC2, Influenza virus

## Abstract

Influenza virus infection is an important cause of severe asthma exacerbations, but it remains unclear how a Th1‐mediated antiviral response triggers a prototypical Th2 disease. We investigated CD4^+^ T cells and group 2 innate lymphoid cells (ILC2s) in influenza virus‐infected mice. We found that ILC2s accumulated in the lung rapidly after influenza virus infection, but the induction of IL‐5 and IL‐13 secretion was delayed and concomitant with T cell activation. In an influenza‐induced exacerbation of allergic airway inflammation model we noticed an initial reduction of ILC2 numbers and cytokine production in broncho‐alveolar lavage compared to chronic house dust mite (HDM)‐mediated airway inflammation alone. ILC2s phenotype was characterized by low T1/ST2, ICOS, KLRG1, and CD25 expression, resembling naïve ILC2s. The contribution of ILC2s to type 2 cytokine production in the early stage of the influenza‐induced exacerbation was limited. In contrast, T cells showed increased IL‐4 and IL‐5 production when exposed to both HDM and influenza virus. Upon virus clearance, ILC2s regained an activated T1/ST2^high^ICOS^high^KLRG1^high^CD25^high^ phenotype paired with cytokine production and were major contributors to the type 2 cytokine milieu. Collectively, our data indicate that both T cells and ILC2s contribute to influenza‐induced exacerbation of allergic airway inflammation, but with different kinetics.

## Introduction

Asthma exacerbations are often provoked by respiratory viruses, particularly rhinovirus and RSV 1, 2. While influenza virus infections occur less often, it is able to cause the most severe forms of asthma exacerbations 3, 4. Adult asthma patients have a higher incidence of influenza virus infection than children, suggesting that adults are more susceptible 5. Influenza virus causes severe epithelial cell damage 6 and induces NF‐κB‐mediated release of cytokines from these cells, such as IL‐1β, IL‐6, and IL‐8 7, 8. This compromises the epithelial barrier function and additionally increases vascular endothelial permeability 9, resulting in a highly robust inflammatory response. In conjunction with the pre‐existing inflammatory environment in asthma patients, loss of barrier function provides a partial explanation for the clinical observations that indicate an association between influenza virus infection and asthma exacerbation. However, the precise underlying pathways remain elusive.

Allergic airway inflammation is centrally mediated by Th2 cells that secrete the key effector cytokines IL‐4, IL‐5, and IL‐13 resulting in characteristic asthma pathology that includes persistent eosinophilic inflammation, airway hyperresponsiveness and remodeling, smooth muscle cell hyperplasia, goblet cell metaplasia, and increased angiogenesis 10, 11. Group 2 innate lymphoid cells (ILC2) also secrete IL‐5 and IL‐13 when stimulated by epithelium‐derived IL‐25, IL‐33, and thymic stromal lymphopoietin (TSLP) as well as IL‐4 under particular conditions 12. Several studies have stressed the potency of ILC2s as a crucial early T cell‐independent source of type 2 cytokines in papain‐ or *Alternaria*‐induced allergic airway inflammation 13, 14, 15, 16. However, we and others have demonstrated that the contribution of T cells is critical in other allergen‐based models of asthma, such as house dust mite (HDM), OVA, and *Aspergillus* 17, 18, 19. Importantly, the groups of Chang et al. and Monticelli et al. have found increased ILC2 numbers in the lungs of influenza virus‐infected mice. Hereby, ILC2s can contribute to airway hyperreactivity, but can also be protective because of their capacity to produce amphiregulin, a member of the epidermal growth factor family that restores epithelial cell integrity 20, 21.

These findings—together with evidence that during influenza virus infection NKT cells and alveolar macrophages can be important endogenous sources of IL‐33 that enhance IL‐5 production from ILC2s 22—highlight a possible role for ILC2s as the causative agent between influenza virus infection and exacerbation of allergic airway inflammation. Moreover, they open up new avenues of research into treatment of severe asthma exacerbations, as the current standard treatment with high doses of inhaled or systemic corticosteroids is not always effective and has a high risk of side effects 23, 24.

When the effect of influenza virus infection on an acute model of HDM‐driven allergic airway inflammation was studied in mice, simultaneous activation of eosinophils and neutrophils and a synergistic effect of influenza and HDM on airway resistance and cell influx into the BALF was found 25. In addition, a marked innate response followed by an accumulation of eosinophils, neutrophils, dendritic cells, and T cells was reported in an X31 H3N2 influenza virus infection model preceded by chronic exposure to HDM 26. In these experiments, anti‐IL‐5 treatment prevented eosinophil influx and influenza‐induced exacerbation of airway inflammation. However, the involvement of ILC2s in the influenza‐mediated exacerbations remains unknown.

Here, we studied the kinetics and function of ILC2s in influenza virus infection and their contribution to influenza‐mediated exacerbation in a robust, physiologically relevant, chronic model of HDM‐induced allergic airway inflammation. We found that although ILC2s accumulated earlier in the lungs than T cells after influenza virus infection, their cytokine production was delayed and only initiated after the peak of T cell activation. The contribution of ILC2s to the total type 2 cytokine milieu in influenza‐mediated exacerbation was modest in the early stages of infection but increased to significant levels at day 11 post infection when the virus was cleared. Because in these experiments also Th2 cytokine production by CD4^+^ T cells was increased, we conclude that both T cells and ILC2s play a role in influenza‐induced exacerbation of allergic airway inflammation, but with different kinetics.

## Results

### Influenza virus infection induces major changes in expressed genes in the lungs

**Figure 1:**
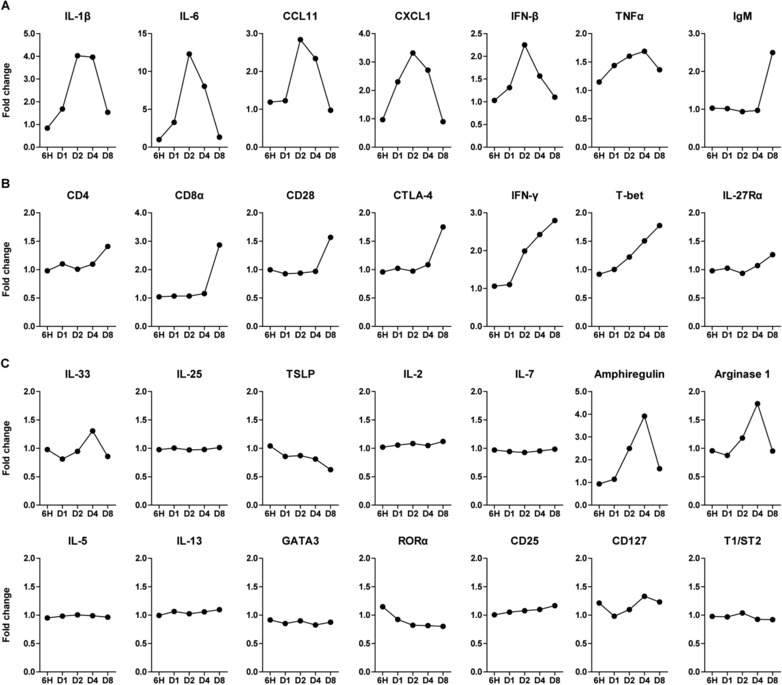
Expression profiles of genes associated with ILC2s and T cells show distinct temporal patterns in influenza virus infection. Quantification of the expression levels of (A) inflammatory mediators, (B) T cell‐related genes, and (C) ILC2‐related genes at 6 h, 1 day, 2, 4, and 8 days after X31 influenza virus inoculation, expressed as fold change compared to PBS‐treated mice (*n* = 5) from a single experiment. Expression was determined by microarray analysis.

We aimed to identify the activation patterns of T cells and ILC2s in mice infected with the X31 H3N2 strain of influenza virus. Gene expression profiling of total lung cells over time revealed distinct patterns for genes associated with innate and adaptive immunity (Fig. 1). Compared to PBS‐treated animals, several pro‐inflammatory mediators exhibited a response that peaked at day 2–4 post infection, indicating initiation of viral immunity. Notably, IL‐1β and IL‐6 were increased ∼four‐ and ∼12‐fold, respectively. Chemokines such as CCL11 and CXCL1 were also upregulated, which attract eosinophils and neutrophils, respectively. Likewise, factors secreted by innate immune cells, including IFN‐β and TNF‐α were elevated early after infection (Fig. 1A). As expected, upregulation of genes that signify an adaptive immune response, such as IgM and T cell‐associated markers (CD4, CD8α, CD28, and CTLA‐4), occurred later in the infection at day 8 (Fig. 1A and B). Hereby, genes associated with Th1 immunity, IFN‐γ, T‐bet, and IL‐27Rα also showed maximal induction at day 8.

Chang et al. have demonstrated rapid increase of IL‐33, a potent activator of ILC2s, in the lungs after influenza virus infection and have shown alveolar macrophages as a potential source 20. Our gene expression analysis confirmed this and we also found an upregulation of IL‐33 at day 4 after inoculation. However, other genes implicated in ILC2 activation including IL‐25, TSLP, IL‐2, and IL‐7, did not follow this pattern. Amphiregulin and arginase‐1, cytokines known to be produced by ILC2s 21, 27, arose concomitantly with the increase in IL‐33 expression. Several signature ILC2 genes, including IL‐5 and IL‐13 cytokines, and the transcription factors GATA3 and RORα remained remarkably unaltered (Fig. 1C). Although these findings may suggest that expression changes of these genes are difficult to detect in total lung, alternatively IL‐33‐driven Th2 cytokine production may be suppressed during influenza infection.

Taken together, this expression analysis shows that influenza virus infection induces major changes in gene expression within the lungs, reflecting induction of innate and adaptive immune responses. Changes in ILC2‐associated genes were not readily detected, but several cytokines that were reported to suppress ILC2s, including type I IFNs and IFN‐γ 28, 29, 30, 31, 32, were clearly induced.

### In influenza virus infection T cells and ILC2s display distinct activation kinetics

**Figure 2:**
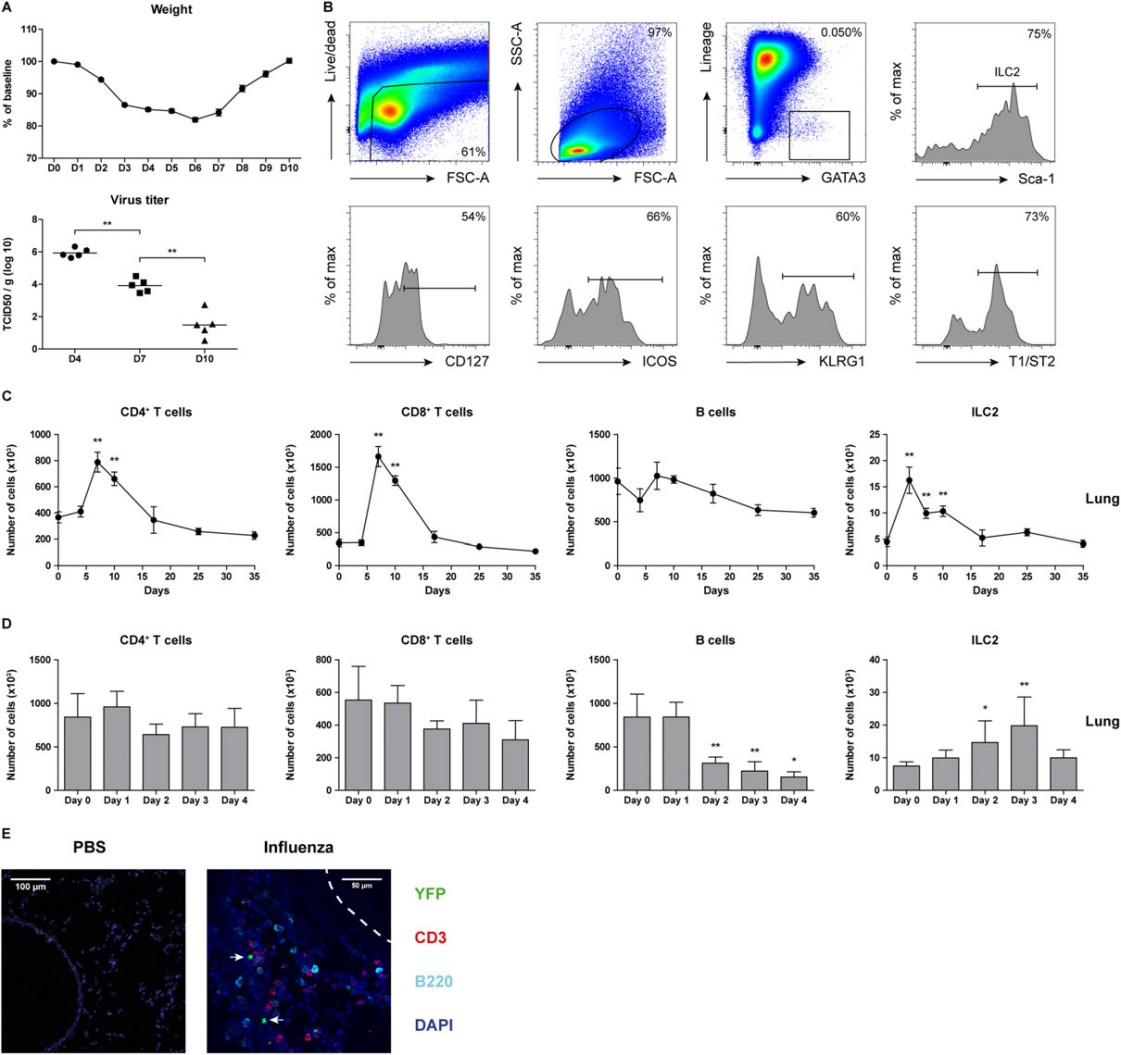
Accumulation of ILC2s in the lungs precedes T cell recruitment after inoculation with influenza virus. (A) Percentage of weight loss in mice infected with 10^4^ PFU X31 influenza virus particles and measurement of virus titers in the lungs at the indicated days post infection. (B) Flow cytometric identification of ILC2s in BALF of X31 virus‐infected mice and surface expression of CD127, ICOS, KLRG1, and T1/ST2. Dot plots showing subsequent gating represent combined data using the concatenate option in FlowJo (*n* = 7) of a single experiment, representative of two independent experiments. (C) Kinetics of CD4^+^ T cells, CD8^+^ T cells, B cells, and ILC2s in the lungs after X31 influenza virus infection. (D) Detailed kinetics of CD4^+^ T cells, CD8^+^ T cells, B cells, and ILC2s in the lungs up to day 4 post infection. (E) Lung cryosection from PBS and X31 virus‐infected *Gata3* reporter (GATIR) mice, counterstained with CD3 (T cells) and B220 (B cells and plasmacytoid dendritic cells). Airway epithelium is outlined by the dashed line. ILC2s, indicated with arrows, are identified as CD3^−^YFP^+^ cells. In the confocal microscopy conditions, GATA3^low^ cells (such as non‐Th2 CD4^+^ T cells, CD8^+^ T cells, and other ILC lineages) are not detectable as YFP^+^ cells. Representative image of *n* = 3 mice per group from three independent experiments. (A, C – D) Data are shown as mean values ± SEM (*n* = 5) of a single experiment from two independent experiments. *p ≤ 0.05, **p ≤ 0.01.

To study the various innate and adaptive immune cell populations during influenza virus infection in detail, we infected mice with 10^4^ PFU viral particles and followed the composition of the immune response over a period of 35 days. Mice rapidly lost weight in the first 4 days, after which their weight stabilized and gradually recovered from day 7 onward (Fig. 2A). Virus titers significantly decreased by day 7 and were almost undetectable by day 10 (Fig. 2A). ILC2s were characterized by flow cytometry as Lineage^−^ lymphocytes that expressed intracellular GATA3 and Sca‐1, as described previously (Fig. 2B) 17. Although expression of the ILC2‐associated markers IL‐7R (CD127), ICOS, KLRG1, and IL‐33R (T1/ST2) was heterogeneous 33, the majority of these cells were positive for these surface markers (Fig. 2B). In agreement with the gene expression data in Fig. 1, increased numbers of CD4^+^ and CD8^+^ T cells were present in the lungs at day 7, after which a slow decline to baseline was reached at day 17. In contrast, ILC2 accumulation was already evident at day 4 post infection and remained significantly elevated in the lungs at day 7 and day 10, and returned to baseline values by day 17 (Fig. 2C). Subsequently, we zoomed in on the initiation phase of the response to influenza virus infection and found that whereas the apex of ILC2 influx into the lungs occurred at day 3, T cell numbers remained unchanged. We observed a slight, but significant reduction in B cell numbers in the lung (Fig. 2D). By employing a *Gata3* reporter mouse, which exhibits concomitant expression of GATA3 and yellow fluorescent protein (YFP) 33 [T.N.R and H.J.F., manuscript in preparation], we were able to visualize the location of ILC2s in the lung during influenza virus infection. ILC2s appeared underneath the airway epithelium, often in close proximity to CD3^+^ T cells (Fig. 2E), also in severe cases where a strong influx of B220^+^ cells (plasmacytoid dendritic cells or B cells) was present (Supporting Information Fig. 1).

Taken together these data indicate that upon influenza virus infection ILC2s in the lung increase more rapidly in number than CD4^+^ or CD8^+^ T cells.

### ILC2 cytokine response to influenza virus follows accumulation in BALF and CD25 upregulation

Given that Th1‐associated cytokines may drive plasticity of ILC2s 34, 35, 36, 37, we examined the expression of cytokines as well as a number of cell surface markers of ILC2s over time in influenza virus‐infected mice.

**Figure 3:**
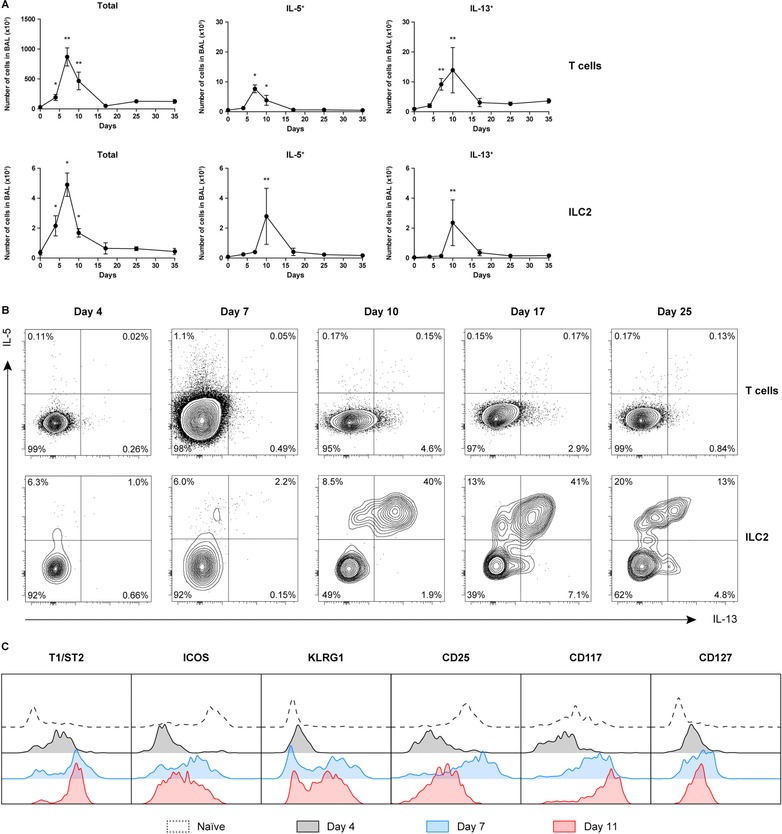
Delayed ILC2 cytokine production in influenza virus infection. (A) Quantification of total IL‐5 and IL‐13 producing T cells and ILC2s in BALF over time after X31 virus infection. Data are shown as mean ± SEM (*n* = 5) of a single experiment from two independent experiments. *p ≤ 0.05, **p ≤ 0.01. (B) Flow cytometric analysis of IL‐5 and IL‐13 expression in BALF T cells and ILC2s at day 4, 7, 10, 17, and 25 after X31 influenza virus infection. (C) Expression of ILC2 surface markers at day 4, 7, and 11. (B, C) Plots represent combined data using the concatenate option in FlowJo (*n* = 5–7) of a single experiment, representative of two independent experiments.

While the numbers of IL‐5^+^ and IL‐13^+^ T cells present in BALF significantly increased at day 7 and 10, only a small proportion of T cells produced these Th2 cytokines (Fig. 3A and B). The numbers of ILC2s in BALF increased as early as day 4 after infection and reached their highest values at day 7, however, type 2 cytokine production by this wave of ILC2s was remarkably limited. Rather, cytokine production by ILC2s was delayed and only initiated in a later phase of influenza virus infection at day 10, when the total number of ILC2s in the BALF was declining (Fig. 3A and B). A comparable pattern, reflecting a peak of IL‐5/IL‐13‐expressing T cells at day 7 and IL‐5/IL‐13‐expressing ILC2s at day 10, was also observed in the lungs and the mediastinal LNs (Supporting Information Fig. S2).

We observed that on the cell surface of ILC2s in the BALF, T1/ST2, KLRG1, and CD117 were upregulated at day 4 and 7 and remained highly expressed at day 11 (Fig. 3C). Interestingly, high IL‐2R (CD25) expression on ILC2s was only observed on day 7, coinciding with the peak of T cell activation, after which it was downregulated. Compared with naïve mice, ICOS expression was downregulated during influenza infection, particularly at day 4 after inoculation (Fig. 3C).

Taken together, these data show that during influenza virus infection the capacity of ILC2s to produce IL‐5/IL‐13 is delayed and is only acquired after their accumulation in the BALF, after upregulation of T1/ST2 and CD25 and after the accumulation of activated T cells.

### Influenza virus infection does not increase ILC2s in the BALF in HDM‐mediated inflammation

**Figure 4:**
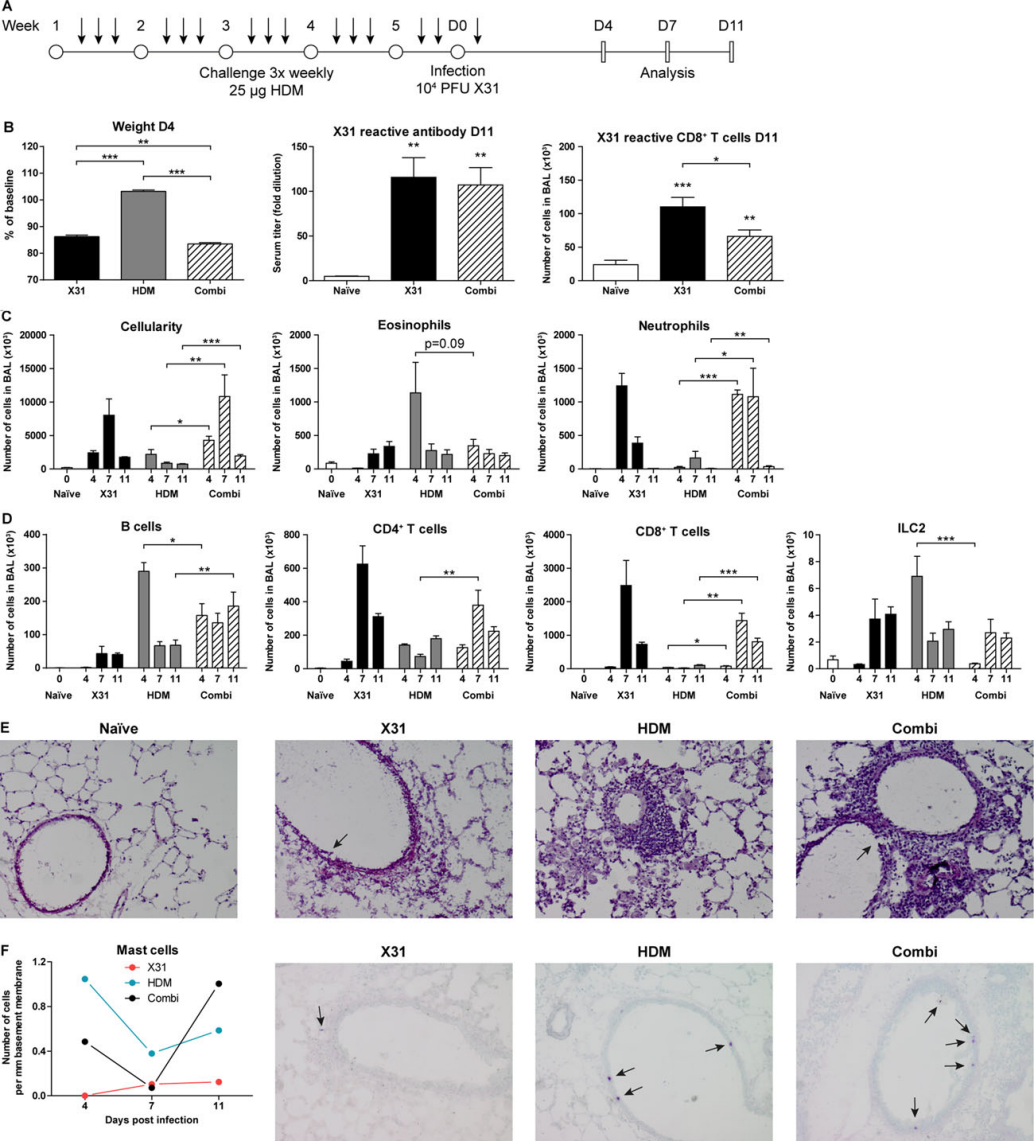
Influenza‐induced exacerbations of allergic airway inflammation are primarily neutrophilic and lead to increased T cell numbers in BALF. (A) Scheme for administration of HDM (arrows) for induction of chronic asthma followed by X31 influenza virus inoculation and analyses at days 4, 7, and 11 post infection. (B) Weight loss of mice inoculated with X31 virus, compared to HDM‐treated mice with or without X31 virus (*left*). X31 influenza specific antibody titer in serum (*middle*) and influenza virus‐specific CD8^+^ T cells in BALF (*right*). (C) Enumeration of total cells, eosinophils, and neutrophils infiltrating in the BALF at day 4, 7, and 11 post infection. (D) Quantification of B cells, CD4^+^ T cells, CD8^+^ T cells, and ILC2s in the BALF. (E) H&E stained cryosections of lungs from naïve, HDM only, X31 virus only, and combination‐treated mice at day 4 post infection; original magnification ×100; arrows indicate epithelial damage. Representative images of *n* = 2 mice per group of a single experiment from two independent experiments. (F) Quantification of intraepithelial mast cells in toluidine blue stained cryosections of lungs from HDM only, X31 influenza virus only, and combination‐treated mice. Data are shown as mean values (*n* = 2) from a single experiment. Representative images of day 11 post infection are displayed from a single experiment (*n* = 2); original magnification ×100; arrows indicate mast cells. (B–D) Data are shown as mean values ± SEM (*n* = 7) of a single experiment from two independent experiments. *p ≤ 0.05, **p ≤ 0.01, ***p ≤ 0.001.

We previously found in chronic HDM‐driven allergic airway inflammation that the numbers of both T cells and ILC2s were significantly increased in the BALF (∼50.000–100.000 and ∼1.000–3.000, respectively). Hereby, ILC2s constituted ∼40% of the IL‐5^+^ lymphocytes and ∼30% of IL‐13^+^ lymphocytes and were mainly CD25^low^ 33, 38. To explore the contribution of ILC2s in influenza‐induced exacerbations of allergic airway inflammation, we developed a mouse model in which we first chronically exposed mice to HDM and subsequently infected them with X31 influenza virus. In these experiments, we included groups of control mice that were either chronically exposed to HDM only or that were infected with influenza virus without prior exposure to HDM. The immune response was analyzed by flow cytometry at day 4, day 7, and day 11 post infection (Fig. 4A).

Mice that received both HDM and X31 virus treatment lost marginally more weight than those only infected with X31 virus (Fig. 4B). Compared to influenza‐only mice, the combination treated animals had similar virus‐specific antibody titers in serum but showed a decrease in the numbers of CD8^+^ T cells that carried a T cell receptor specific for the X31 epitope NP_366‐374_ (ASNENMETM) in the BALF (Fig. 4B). Importantly, the combination treatment resulted in a significantly higher influx of immune cells into the BALF than HDM alone at all time points investigated (flow cytometric gating strategy illustrated in Supporting Information Fig. S3). The infiltrating granulocytes in the combination group consisted predominantly of neutrophils, but a significant eosinophil presence was also observed. The total influx of cells in the BALF peaked at day 7, although both eosinophil and neutrophil numbers remained comparable to the levels seen at day 4. By day 11, neutrophils returned to baseline levels and the inflammation reached its resolution phase (Fig. 4C). The combination of HDM exposure and X31 virus infection led to increased numbers of B cells, CD4^+^, and CD8^+^ T cells compared to HDM only, particularly at day 7 or 11 (Fig. 4D).

Notably, X31 influenza virus infection significantly reduced ILC2 numbers in HDM‐treated mice at day 4 to <1000 cells, despite a strong CD4^+^ T cell presence, and recovered to a similar range as HDM‐treated group by day 7 (Fig. 4D). Examination of lung cryosections stained with H&E confirmed a more severe inflammatory response in the combination treatment around the airways, compared to HDM alone and showed signs of epithelial damage similar to X31 virus infection alone (shown for day 4 in Fig. 4E). Mast cells were detected within the epithelial layer in mice chronically exposed to HDM but not after X31 virus infection alone, where only very low numbers of mast cells resided underneath the basement membrane. Interestingly, upon infection of HDM‐treated mice with X31 influenza virus, the numbers of intraepithelial mast cells decreased and were almost undetectable by day 7. Mast cells reemerged in the epithelium after virus clearance by day 11 (Fig. 4F).

In summary, influenza virus infection following HDM‐mediated allergic airway inflammation differentially affects CD4^+^ T cells and ILC2s and induces a transient reduction of ILC2 numbers in the BALF at day 4, compared to HDM‐mediated inflammation only.

### Different kinetics of cytokine response by T cells and ILC2s in exacerbation of airway inflammation

**Figure 5:**
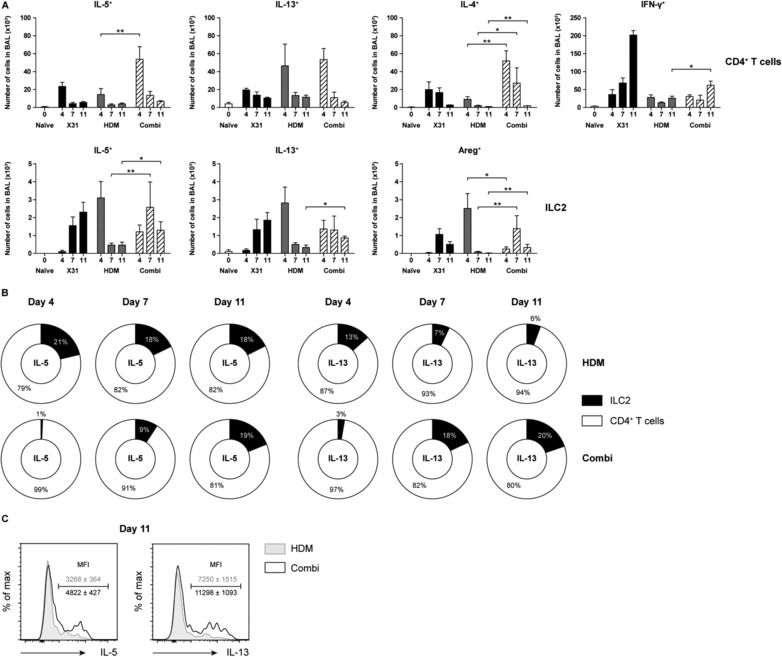
ILC2s are important contributors to type 2 cytokine producers in the late phase of influenza‐induced exacerbations of allergic airway inflammation. (A) Quantification of the absolute number of cytokine producing CD4^+^ T cells (top) and ILC2s (bottom). (B) Ratio of IL‐5 and IL‐13 producing ILC2s and CD4^+^ T cells over time in BALF. (C) Flow cytometric comparison of IL‐5 and IL‐13 production by BALF ILC2s at day 11 post infection. Histogram overlay plots represent combined data using the concatenate option in FlowJo (*n* = 7) of a single experiment, representative of two independent experiments. (A–C) Data are shown as mean ± SEM (*n* = 7) of a single experiment representative of two independent experiments. *p ≤ 0.05, **p ≤ 0.01.

Next, we used intracellular flow cytometry to evaluate the capacity of Th2 cells and ILC2 to produce cytokines. These analyses revealed that the numbers of IL‐4^+^ and IL‐5^+^ CD4^+^ T cells were significantly enhanced at day 4 and 7 of the X31‐induced exacerbation of HDM‐mediated allergic airway inflammation, when compared to influenza virus infection or HDM‐challenge alone (Fig. 5A). Interestingly, the numbers of IL‐5^+^, IL‐13^+^, and amphiregulin‐expressing ILC2s were increased at day 7 and day 11 but not at day 4, as compared to HDM alone (Fig. 5A). The additive effect of X31 virus on IL‐4^+^ CD4^+^ T cells and IL‐5^+^ ILC2s was also observed in the lungs (Supporting Information Fig. S4).

Overall, when we compared the relative contribution of CD4^+^ T cells and ILC2s to the population of type 2 cytokine expressing cells, it was apparent that CD4^+^ T cells were the major source of IL‐5 or IL‐13 in the early phase of influenza‐induced exacerbation (Fig. 5B). At later time points following influenza virus infection, however, ILC2s contributed more to the total number of IL‐5 and IL‐13 producing cells, although CD4^+^ T cells remained dominant (Fig. 5B). Moreover, the MFI values for IL‐5 and IL‐13 content in ILC2s at day 11 was significantly higher in combination‐treated mice, indicating a higher cytokine production capacity per cell (Fig. 5C).

Taken together, these data imply that both T cells and ILC2s are major effector cells that contribute to the type 2 cytokine milieu in influenza‐induced exacerbation of allergic airway inflammation, albeit with different kinetics.

### ILC2s display an activated phenotype only after virus clearance

Finally, we compared the surface phenotype of ILC2s as an indicator of the activation status of ILC2s in chronic HDM‐driven allergic airway inflammation, with and without X31 influenza virus‐induced exacerbation, as described in Fig. 4A.

**Figure 6:**
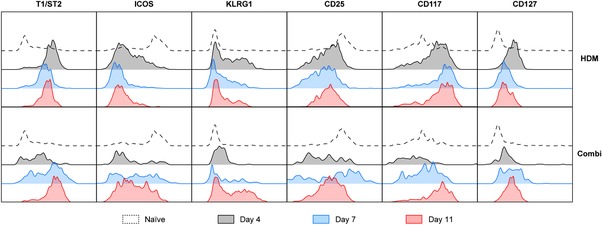
Influenza virus infection initially suppresses HDM‐activated ILC2s but induces enhanced activation at day 11. Flow cytometric comparison of T1/ST2, ICOS, KLRG1, CD25, CD117, and CD127 on ILC2s derived from BALF at day 0, 4, 7, and 11. Histogram overlay plots represent combined data using the concatenate option in FlowJo (*n* = 7) of a single experiment, representative of two independent experiments. Quantification of these data is given in Supporting Information Fig. S5.

We noticed upregulation of T1/ST2 and KLRG1 paired with a downregulation of ICOS and CD25 in HDM‐treated mice compared to naïve mice at day 4 (Fig. 6; quantified in Supporting Information Fig. S5). As expected, the activated phenotype appeared to diminish slowly over time, as T1/ST2 and KLRG1 surface expression decreased, but did not yet return to baseline levels at day 11. In the combination group, however, surface T1/ST2 and KLRG1 expression remained close the levels found in naïve mice, suggesting that X31 influenza virus infection resulted in suppression of ILC2s that were activated by the HDM exposure (Fig. 6; quantified in Supporting Information Fig. S5).

However, at day 7–11 ILC2s in combination‐treated mice regained expression of various surface markers. Most interestingly, this resulted in an activated T1/ST2^high^ICOS^high^KLRG1^high^CD25^high^ surface phenotype of BALF ILC2s, which was significantly different from the phenotype in mice that were not infected with influenza virus (Fig. 6; quantified in Supporting Information Fig. S5).

Taken together, these findings show that after clearance of the virus by day 11, ILC2s present in the BALF manifest—concomitant with a high capacity to produce type 2 cytokines—an activated surface phenotype.

## Discussion

Virus infection‐induced exacerbations account for a major portion in healthcare costs associated with severe asthma 39. However, the current knowledge of how a Th1 response is able to trigger a Th2‐mediated disease is fragmented. IL‐33 and the type 2 cytokines IL‐4, IL‐5, and IL‐13 have been shown to be induced by rhinovirus infection with a correlation between disease severity and cytokine levels. Furthermore, in vitro data show that supernatant from rhinovirus‐infected bronchial epithelial cells induces type 2 cytokine production in both T cells and ILC2s 40. ILC2s have also been hypothesized to be the missing link in the underlying mechanisms between influenza virus infection and asthma exacerbation due to their accumulation in the lung after influenza virus infection and their ability to secrete type 2 cytokines to promote asthma symptoms 21.

Influenza‐induced asthma exacerbation models have been reported, but the contribution of ILC2s is still unclear 25, 26. In our study we present a model for influenza‐induced exacerbation of allergic airway inflammation in which mice were chronically exposed to HDM 38 and subsequently infected with X31 influenza virus to induce exacerbation of airway inflammation. Using multicolor flow cytometry, we observed an increased cellularity of the BALF consisting of a mixed eosinophilic and neutrophilic infiltrate in influenza‐infected HDM‐exposed mice, compared to major eosinophilic inflammation present in mice that were only exposed to HDM. Concomitantly, at day 4 of the influenza virus infection, ILC2 numbers and their cytokine production capacity in BALF were suppressed. However, once the virus was cleared, BALF ILC2s resumed production of type 2 cytokines and gained an activated T1/ST2^high^ICOS^high^KLRG1^high^CD25^high^ surface phenotype on day 11, which was significantly different from the phenotype in mice that were exposed to HDM only. In contrast, T cells in the BALF and mediastinal LNs of HDM‐exposed mice showed particularly at day 4 of the influenza virus infection increased IL‐4 and IL‐5 production, compared to mice that were not infected with influenza virus. Therefore, CD4^+^ T cells in this virus‐induced HDM‐driven inflammation exacerbation model were a more prominent contributor to the type 2 cytokine environment than ILC2s early after influenza virus infection. We conclude that both T cells and ILC2s contribute to influenza‐induced exacerbation of allergic airway inflammation, but with remarkably different kinetics.

Irrespective of the presence of chronic HDM‐driven inflammation, we found that during influenza virus infection ILC2s are induced in the BALF with remarkable kinetics: numbers of ILC2s peak at day 4–7 but substantial IL‐5 and IL‐13 cytokine production capacity was only detected later during infection at around day 10–11 in the resolution phase of influenza virus infection when virus particles had been eradicated and the type 1 immune response was reaching its conclusion. This would be in agreement with the proposed restorative functions of ILC2s 21, but also indicates that an ongoing influenza virus infection significantly suppresses ILC2 activation. This was also evident in our combination treatment in which the numbers of HDM‐activated ILC2s were very low at day 4 after influenza virus infection. A likely explanation for this is that type 1 IFN, IFN‐γ, and IL‐27, which are abundantly present in influenza virus infections (Fig. 1), are able to inhibit ILC2s in a dose‐dependent manner 28, 29, 31, 32. In particular, it was very recently reported that virus‐induced IFN‐γ restricts protective ILC2 functions in the lung following challenge with the pandemic H1N1 CA04 influenza virus, whereby neutralizing IFN‐γ protected mice from lethal infection 30. Our finding that at day 4 of infection both IFN‐β and IFN‐γ are increased supports the hypothesis that these cytokines inhibit ILC2 induction. Other known cytokines that have an inhibitory effect on ILC2 function where either not significantly induced by the influenza virus infection (IFN‐α) or not represented in the Affymetrix mouse microarray 430.2 (IL‐27). It is very well possible that these cytokines act in concert to suppress ILC2 activity. It must be noted however, that different strains of influenza virus may result in alternative ILC2 kinetics. Comparison between the low‐pathological H3N2 X31 strain and the high‐pathological H1N1 PR8 strain show that a more robust CD8^+^ T cell response is generated upon infection with the X31 influenza virus and is paired with higher levels of IL‐12 in the microenvironment and more IFN‐γ production by CD8^+^ T cells 41. Therefore in high‐pathological influenza virus infections such as the PR8 strain, the reduction in IFN‐γ levels as well as more severe epithelial damage may trigger earlier activation of ILC2s.

Nevertheless, upon virus clearance, ILC2s regained a T1/ST2^high^ICOS^high^KLRG1^high^CD25^high^ phenotype paired with cytokine production and were major contributors to the type 2 cytokine milieu. Several lines of evidence support the notion that this surface phenotype signifies strong activation of ILC2s. First, high cell surface expression of T1/ST2 on ILC2s will increase their activation status because IL‐33, produced by epithelial cells, e.g., upon allergen activation, is one of the most potent activators of ILC2s 42, 43, 44. In this context, we previously showed that although in HDM‐mediated airway inflammation the accumulation of ILC2s in BALF is IL‐33 independent, IL‐33 is critical for cytokine production by ILC2s 17. In our combination treatment model, next to HDM‐activated epithelial cells, NKT cells might be an additional source of IL‐33, given that NKT cells were shown to secrete IL‐33 on day 7–12 following infection with H1N1 PR8 influenza virus 22. Alternatively, alveolar macrophages are also a source of IL‐33 in H3N1 Mem/71 influenza virus‐infected mice where ILC2 activation peaks at day 6–9 post infection 20. Second, IL‐2 and IL‐7, which alone are not sufficient for ILC2 activation, are thought to enhance the in vivo effect of IL‐33 13, 45. Therefore, high expression of IL‐2R (CD25) and IL‐7R (CD127)—together with high T1/ST2 expression—would optimally prime ILC2s for receiving activating signals from NKT cells, alveolar macrophages, or epithelial cells via IL‐33. Rapid downregulation of CD25 on ILC2s by day 11 is evidence for tight regulation of this pathway and was followed by a diminishing IL‐5/IL‐13 profile. Third, ICOS:ICOS‐ligand interaction promotes cytokine production, proliferation, and survival of ILC2s both under homeostatic and inflammatory conditions 46, 47 and moreover, ICOS on ILC2s may interact with B7RP‐1‐expressing dendritic cells in allergic inflammatory lung, which increases ILC2 activation 48. Finally, KLRG1 has been reported as an ILC2 activation marker and is intermediate to highly expressed by mature ILC2s 49, 50. Heterogeneous surface expression of CD117 on ILC2s during an influenza virus infection has been previously described. Because the authors did not observe functional differences between CD117^−^ and CD117^+^ ILC2s, they concluded that the marked accumulation of CD117^+^ ILC2s during the recovery phase of influenza virus infection most likely reflects recruitment of these cells from the BM 22. In this context, it is important to note that our T1/ST2^high^ICOS^high^KLRG1^high^CD25^high^ cells are different from the KLRG1^high^ inflammatory ILC2 population that is responsive to IL‐25 and is proposed to be transient progenitors mobilized by inflammation 49. In contrast to these cells, which do not express T1/ST2, our activated ILC2s are T1/ST2^high^.

Our comparison of chronic HDM‐driven allergic airway inflammation, with and without X31 influenza virus‐induced exacerbation (day 11), indicated a correlation between the presence of mast cells and ILC2 activation. Interestingly, mast cell‐derived mediators, such as IL‐2, IL‐33, prostaglandin D_2_, and leukotriene E_4_, enhance ILC2 responses 51, 52, 53 and serine proteases secreted by activated mast cells cleave IL‐33 into mature and more biologically active forms 54. Conversely, IL‐9 production by ILC2 can induce mast cell hyperplasia 55, leading to a positive feedback loop. Therefore, it is conceivable that mast‐cell ILC2 interaction contributes to the increased activation of ILC2s in the recovery phase of influenza infection in our exacerbation model.

In summary, our mouse model of influenza‐induced exacerbation of chronic HDM‐driven allergic airway inflammation presents a mixed eosinophilic and neutrophilic infiltrate in the BALF and an initial suppression of ILC2 accumulation. After virus clearance, however, ILC2s are significant cytokine producers and show an activated T1/ST2^high^ICOS^high^KLRG1^high^CD25^high^ state, which would prime ILC2s for receiving activating signals from allergen‐activated epithelial cells. It is attractive to speculate that these activated ILC2s share characteristics with the allergen‐experienced ILC2s that persist after resolution of inflammation and were shown to respond more potently to unrelated allergens than naïve ILC2s did 56. It was proposed that memory‐like properties of allergen‐experienced ILC2s may explain why asthma patients are often sensitized to multiple allergens. Likewise, it is conceivable that—next to T cells—memory‐like properties of ILC2s that are induced upon viral infections may contribute to exacerbations of airway inflammation upon allergen exposure. Furthermore, type 2 cytokines such as those secreted by ILC2s possess pro‐fibrotic functions through activation of fibroblasts and may result in additional remodeling of the airways 57, 58.

## Materials and methods

### Mice

Wildtype C57BL/6 mice were purchased from Envigo (United Kingdom). Homozygous *Gata3* reporter (GATIR) mice, which harbor an IRES‐YFP sequence within the 3’ untranslated region of the *Gata3* gene resulting in concomitant production of GATA3 and YFP protein, were bred on a C57BL/6 background [T.N.R. and H.J.F., manuscript in preparation; Stadhouders et al., manuscript in revision]. Mice were ∼8–16 weeks old at the time of analysis. All animals were housed at the Erasmus MC Animal Facility under SPF conditions. All experiments were approved by the Erasmus MC Animal Ethics Committee.

### Induction of chronic allergic airway inflammation

To induce chronic allergic airway inflammation, mice were anesthetized using isoflurane and treated intranasally three times per week for five consecutive weeks with 25 μg HDM extract (Greer, USA) or PBS (GIBCO Life Technologies, USA) as previously described 38.

### X31 influenza virus infection and gene expression profiling

Inoculation and harvest of the X31 H3N2 strain of influenza virus (Medical Research Council, UK) has been described previously 59. Mice were anesthetized using isoflurane and infected with 10^4^ PFU X31 particles diluted in PBS via intranasal inoculation. Animals were sacrificed after infection at the indicated time points. Virus titers in lung tissue were determined as previously described 59, 60 X31‐reactive antibody titers in serum was detected by hemagglutination inhibition assay as reported in the World Health Organization manual for laboratory diagnosis and virologic surveillance of influenza 61.

Gene expression profiling was performed in mice infected with 10^5^ PFU X31 virus particles as described elsewhere 62. In brief, the lungs were excised and stored in RLT buffer (Qiagen, Germany) at –80°C. Total RNA was then isolated using an RNeasy kit (Qiagen, Germany) and biotin labeled and hybridized to mouse DNA microarrays (Affymetrix, USA).

### Flow cytometry

Single cell suspensions were prepared from lungs and lymph nodes by mechanical disruption without digestive enzymes using a 100 μm cell strainer (BD Biosciences, USA) in PBS supplemented with 0.5% BSA and 5 mM EDTA (Sigma–Aldrich, USA). For determination of intracellular cytokine content, cells were stimulated with PMA and ionomycin (Sigma–Aldrich, USA) at 37°C for 4 h prior to staining. Flow cytometry surface and intracellular staining was performed as described previously 44, 63. In brief, intracellular transcription factors were stained by fixation and permeabilization using eBioscience™ FOXP3/transcription factor staining buffer set (Thermo Fisher Scientific, USA) followed by 1 h incubation with fluorescent antibodies at 4°C. Intracellular cytokines were stained by fixation with 2% PFA (Thermo Fisher Scientific, USA) and permeabilization with 0.5% saponin (Sigma–Aldrich, USA) followed by 1 h incubation with fluorescent antibodies at 4°C. Dead cells were excluded using LIVE/DEAD™ fixable aqua dead cell stain kit (Thermo Fisher Scientific, USA) and only single cells were gated for further analysis using FSC‐W and FSC‐A. Lineage markers included CD3, CD4, CD5, CD8α, CD11b, CD11c, CD19, B220, NK1.1, FcεRIα, TER‐119, and Gr‐1. X31‐reactive CD8^+^ T cells were detected using MHC dextramer against epitope NP_366‐374_ (ASNENMETM). A comprehensive list of antibodies used for flow cytometry is presented in Supporting Information Table S1. Samples were acquired using a LSR II flow cytometer and FACSDiva (BD Biosciences, USA) and analyzed by FlowJo X (Tree Star Inc., USA). Gating strategy is shown in in Fig. 2 and Supporting Information Fig. S3.

## Histology

Lungs were inflated with OCT embedding medium (Sakura, the Netherlands) diluted in equal volume of PBS prior to being snap frozen in liquid nitrogen. Lungs from *Gata3* reporter mice were inflated with a 1:1 mixture of OCT embedding medium and PFA to preserve YFP fluorescence. Cryosections (7 μm) were cut at –20°C using a cryostat (Thermo Fisher Scientific, USA) and fixed in 4% PFA for 10 min. Then slides were stained with primary antibodies for 1 h and secondary antibodies for 30 min at room temperature. A comprehensive list of antibodies used for confocal microscopy is presented in Supporting Information Table S2. Slides were incubated with DAPI (Invitrogen, USA) for 5 min and sealed with Vectashield (Vector Laboratories, USA). An LSM510Meta confocal microscope equipped with 405, 488, 543, and 633 nm lasers (Zeiss, Germany) was used to examine the slides. Images were processed and analyzed in Fiji, an open source scientific image processing application based on ImageJ.

For H&E stain, cryosections (7 μm) were fixed by dipping in 4% PFA followed by 30 s in Gill's Haematoxylin and 1 min in 4% Eosin B acetified with glacial acetic acid (Sigma–Aldrich). Samples were dehydrated by sequential rinsing in ethanol up to 100% and cleared in xylene for 2 min (Sigma–Aldrich, USA). Sections were mounted in Entallan (Merck, USA) and examined under a light microscope (Zeiss, Germany).

The presence of intraepithelial mast cells was analyzed by staining cryosections (6 μm) with toluidine blue (Sigma–Aldrich) at pH 0.5 for 1–2 min, which were immediately examined under a light microscope. Quantification was performed by counting the number of mast cells per mm basement membrane in bronchioles of the lower airways.

## Statistical analysis

Mann–Whitney *U* tests were used for statistical comparisons between groups and a *p*‐value <0.05 was considered statistically significant. All analyses were performed using Prism 5 (GraphPad Software, USA).

## Conflict of interest

The authors declare no financial or commercial conflict of interest.

**Abbreviations**
HDM: house dust mite
ILC2: group 2 innate lymphoid cell
YFP: yellow fluorescent protein

## Supporting information

Peer review correspondence

Supporting Information
