# Supplementary material for: T cells and ILC2s are major effector cells in influenza‐induced exacerbation of allergic airway inflammation in mice
Source: Eur J Immunol. 2018 Jun 11;49(1):144–56. doi: 10.1002/eji.201747421 (PMC6585726; doi:10.1002/eji.201747421)
Supplement: Supplementary file 2 — Supporting Information [file EJI-49-144-s002.docx]

**Supplementary information to:**

**T cells and ILC2s are major effector cells in influenza-induced exacerbation of allergic airway inflammation**

Bobby W.S. Li^1^, Marjolein J.W. de Bruijn^1^, Melanie Lukkes^1^, Menno van Nimwegen^1^, Ingrid M. Bergen^1^, Corine H. GeurtsvanKessel^2^, Alex KleinJan^1^, Arno Andeweg^2^, Guus F. Rimmelzwaan^2^ and Rudi W. Hendriks^1^

^1^Department of Pulmonary Medicine, Erasmus MC Rotterdam, Rotterdam, the Netherlands

^2^Department of Viroscience, Erasmus MC Rotterdam, Rotterdam, the Netherlands


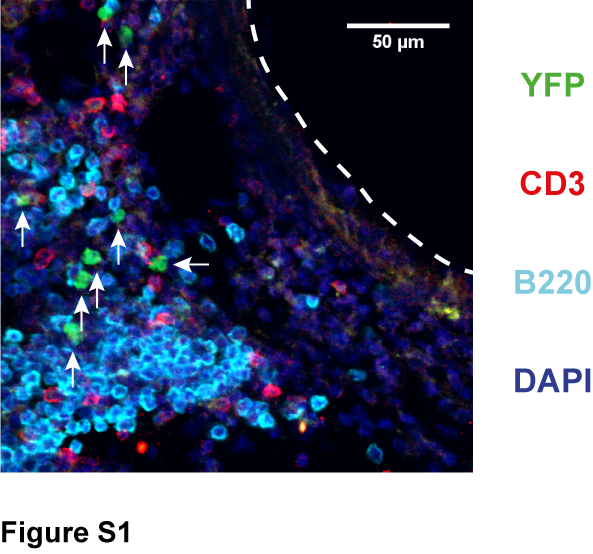


**Figure S1. Severe inflammatory response to influenza recruits T cells, B220^+^ cells and ILC2s at day 4 post infection.**

Lung cryosection from X31 influenza virus infected *Gata3* reporter mice counterstained with CD3 (T cells) and B220 (B cells and plasmacytoid dendritic cells). Airway epithelium is outlined by the dashed line. ILC2s, indicated with arrows, are identified as CD3^-^YFP^+^ cells. In the confocal microscopy conditions, GATA3^low^ cells (such as non-Th2 CD4^+^ T cells, CD8^+^ T cells and other ILC lineages) are not detectable as YFP^+^ cells. Representative image of n = 3 mice per group from three independent experiments.


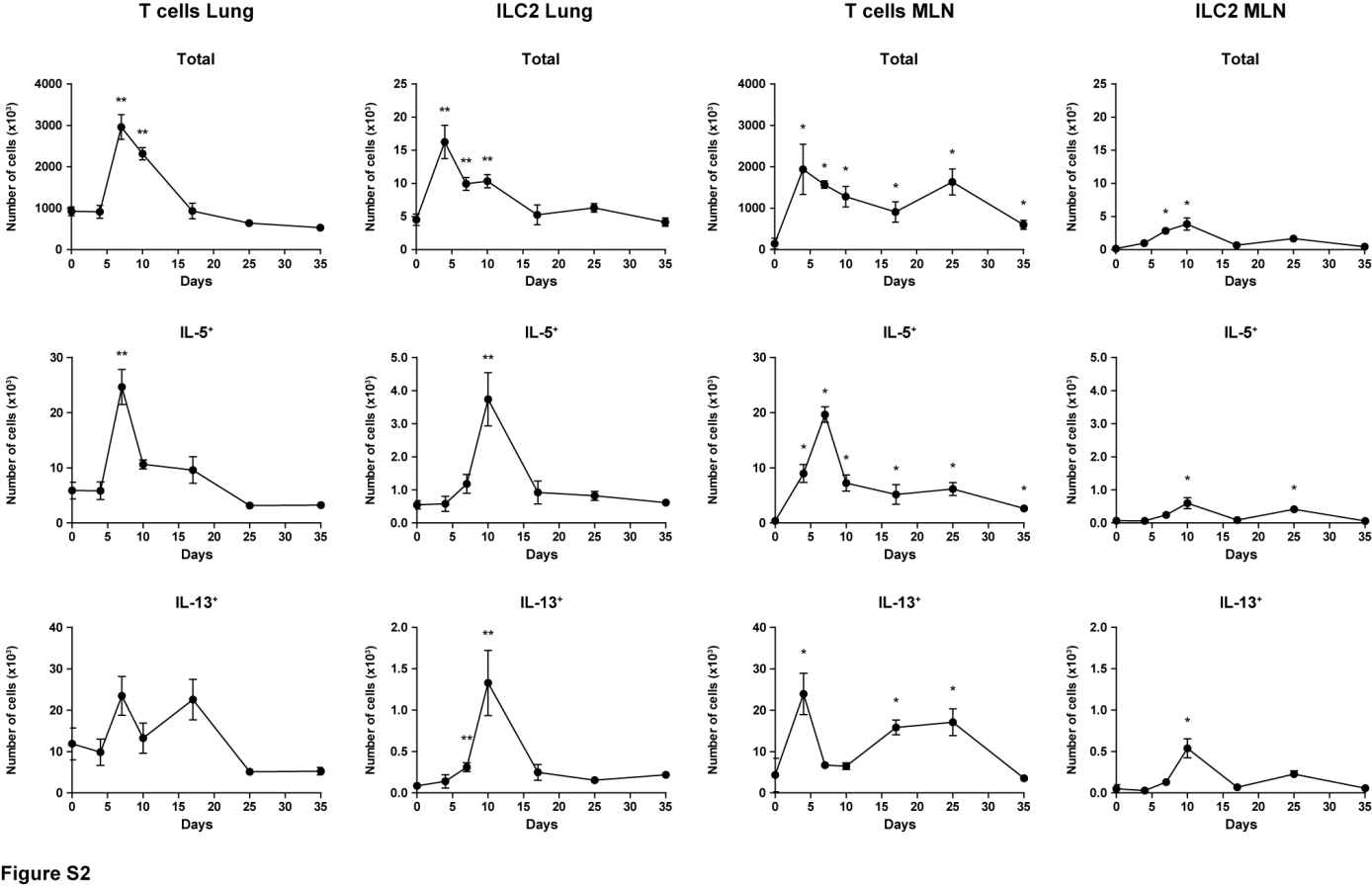


**Figure S2. Kinetics of type 2 cytokine production by ILC2s and T cells after influenza virus infection.**

Quantification of the number of IL-5 and IL-13-producing T cells and ILC2s in the lungs and MLN of mice infected with X31 influenza virus. Data are shown as mean values ± SEM (n = 5) of a single experiment from two independent experiments; * p ≤ 0.05, ** p ≤ 0.01.


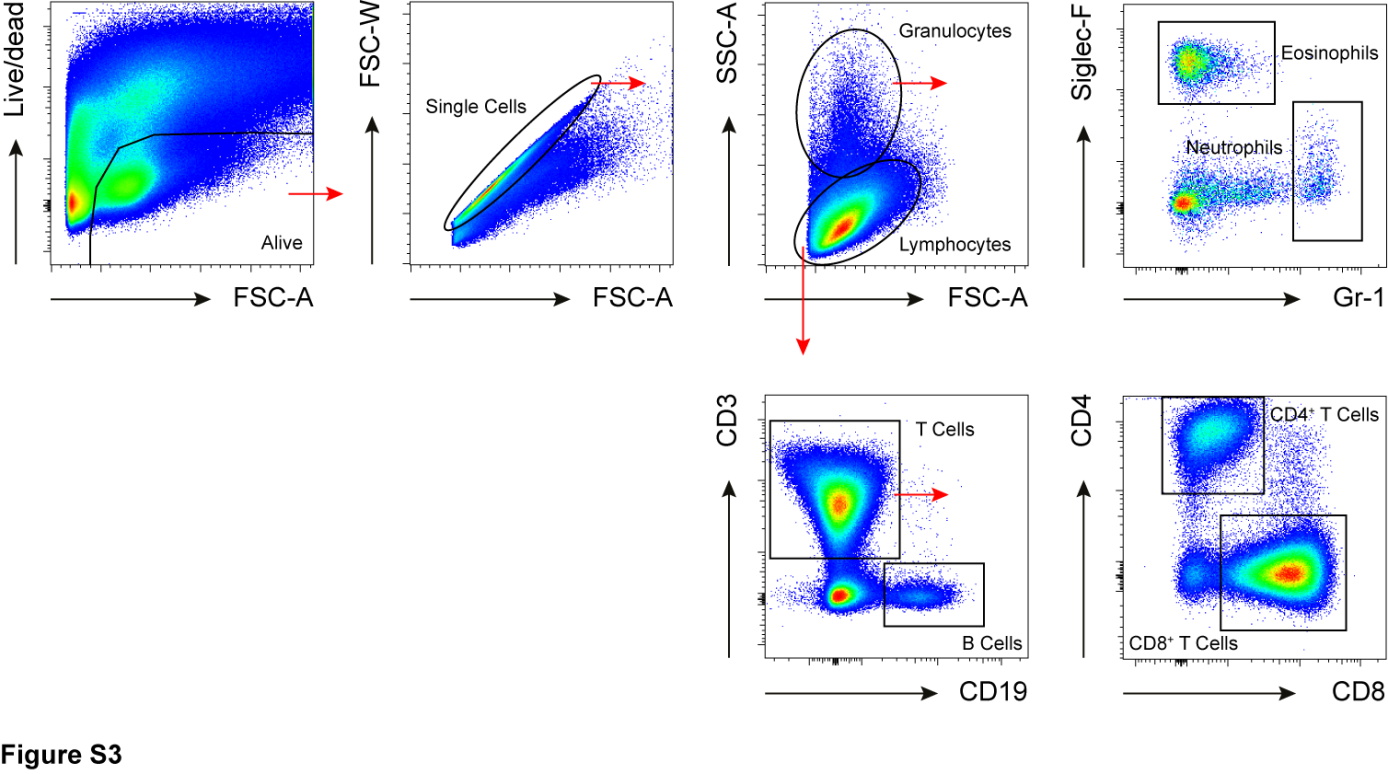


**Figure S3. Flow cytometric analysis of eosinophils, neutrophils, B cells, CD4^+^ T cells and CD8^+^ T cells.**

Plots represent combined data from BAL fluid of HDM-treated and X31 virus-infected mice using the concatenate option in FlowJo (n = 7) of a single experiment, representative of multiple independent experiments.

**
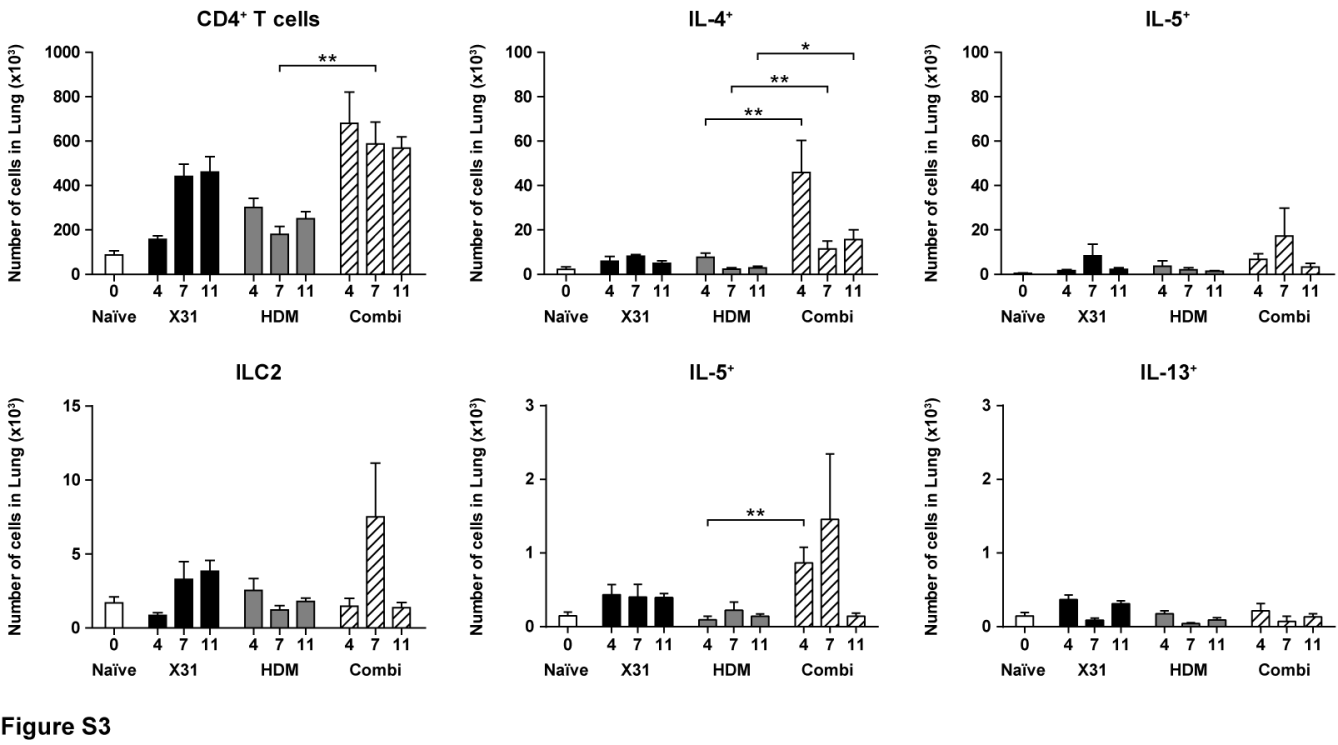
**

**Figure S4. Synergistic effect of influenza virus infection in lungs of HDM-treated mice.**

Quantification of the number of type 2 cytokine producing CD4^+^ T cells (*top*) and ILC2s (*bottom*) in lungs. Not that there are significant synergistic effects of influenza virus infection on the induction of IL-4^+^ T cells and IL-5^+^ ILC2s in the lung. Data are shown as mean values ± SEM (n = 7) of a single experiment from two independent experiments. * p ≤ 0.05, ** p ≤ 0.01.


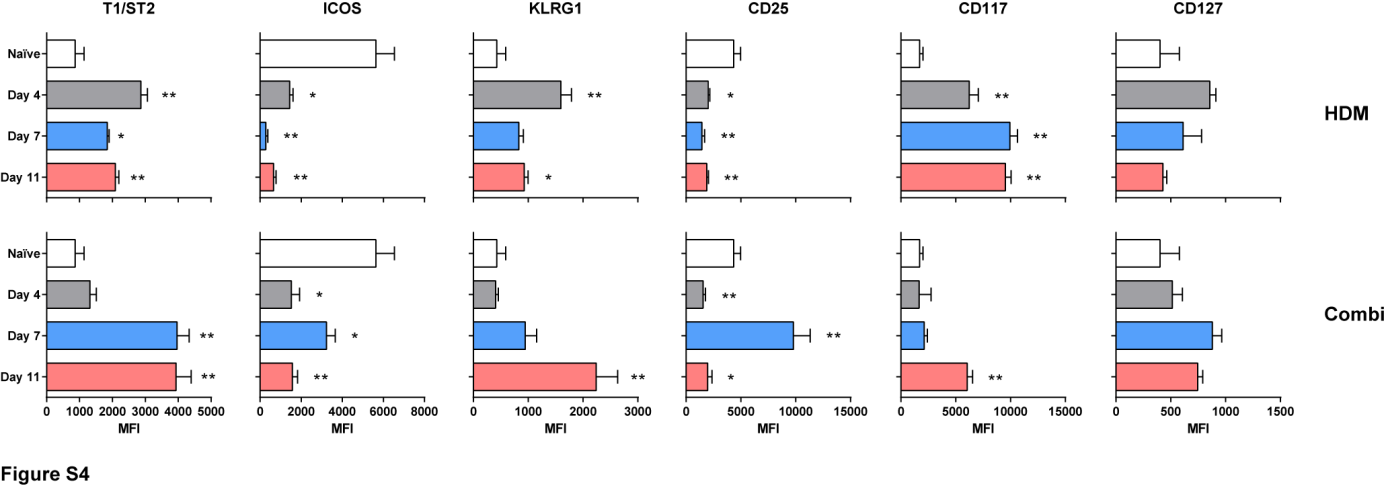


**Figure S5. Quantification of surface expression of T1/ST2, ICOS, KLRG1, CD25, CD117 and CD127 on ILC2s in BAL fluid at day 0, day 4, day 7 and day 11 post influenza infection.**

Mean fluorescence intensity (MFI) values corresponding to histograms shown in Figure 6. Data are shown as mean values ± SEM (n = 7) of a single experiment from two independent experiments. * p ≤ 0.05, ** p ≤ 0.01.

Table S1 Antibodies used for flow cytometry.

| **Antibody** | **Conjugate** | **Clone** | **Company** |
| --- | --- | --- | --- |
| Amphiregulin | Biotin | Polyclonal | R&D Systems |
| B220 | PE | RA3-6B2 | eBioscience |
| CD117 | Brilliant Violet 650 | 2B8 | BD Biosciences |
| CD11b | PE | M1/70 | eBioscience |
| CD11b | PerCP-Cy5.5 | M1/70 | BD Biosciences |
| CD11b | Alexa Fluor 700 | M1/70 | eBioscience |
| CD11c | PE | N418 | eBioscience |
| CD11c | PE-Texas Red | N418 | Invitrogen |
| CD11c | eFluor 450 | N418 | eBioscience |
| CD127 | PE-Cy7 | A7R34 | eBioscience |
| CD127 | eFluor 450 | A7R34 | eBioscience |
| CD19 | PE | 1D3 | BD Biosciences |
| CD19 | PerCP-Cy5.5 | eBio1D3 | eBioscience |
| CD19 | Biotin | 1D3 | BD Biosciences |
| CD19 | Alexa Fluor 700 | eBio1D3 | eBioscience |
| CD25 | PerCP-Cy5.5 | PC61.5 | eBioscience |
| CD25 | Brilliant Violet 605 | PC61 | BioLegend |
| CD3 | PE | 145-2c11 | eBioscience |
| CD3 | PE-CF594 | 145-2c11 | BD Biosciences |
| CD4 | Alexa Fluor 700 | GK1.5 | eBioscience |
| CD4 | PerCP-Cy5.5 | RM4-5 | eBioscience |
| CD4 | Brilliant Violet 605 | RM4-5 | BD Biosciences |
| CD4 | Brilliant Violet 711 | RM4-5 | BD Biosciences |
| CD45 | PE-CF594 | I3/2.3 | Abcam |
| CD45 | Pe-Cy7 | 30-F11 | eBioscience |
| CD5 | PE | 53-7.3 | eBioscience |
| CD8 | APC | 53-7.3 | eBioscience |
| CD8 | APC-EF780 | 53-7.3 | eBioscience |
| CD8 | PE | 53-6.7 | eBioscience |
| CD86 | PE-Cy7 | GL1 | BD Biosciences |
| CD90.2 | FITC | 53-2.1 | BD Biosciences |
| FcεRIα | PE | MAR-1 | eBioscience |
| FoxP3 | Alexa Fluor 700 | FJK-16s | eBioscience |
| FoxP3 | PE-Cy7 | FJK-16s | eBioscience |
| Gata3 | eFluor 660 | TWAJ-14 | eBioscience |
| Gr-1 | PE | RB6-8C5 | BD Biosciences |
| Gr-1 | APC-eFluor 780 | RB6-8C5 | eBioscience |
| ICOS | APC | C398.4A | eBioscience |
| ICOS | PE-Cy7 | 7E.17G9 | eBioscience |
| IFN-γ | APC | XMG1.2 | BD Biosciences |
| IFN-γ | Brilliant Violet 650 | XMG1.2 | BD Biosciences |
| IL-10 | PerCP-Cy5.5 | JES5-16E3 | eBioscience |
| IL-13 | eFluor 450 | eBio13A | eBioscience |
| IL-13 | eFluor 660 | eBio13A | eBioscience |
| IL-17 | Alexa Fluor 700 | TC11-18H10.1 | BD Biosciences |
| IL-4 | Brilliant Violet 711 | 11B11 | BD Biosciences |
| IL-4 | Biotin | BVD4-1D11 | eBioscience |
| IL-4 | PE-Cy7 | 11B11 | BD Biosciences |
| IL-5 | APC | TRFK-5 | BD Biosciences |
| IL-5 | Biotin | TRFK4 | BD Biosciences |
| IL-9 | PerCP-Cy5.5 | D9302C12 | BD Biosciences |
| IL-9 | PE | D9302C12 | BD Biosciences |
| Ki-67 | FITC | SolA15 | eBioscience |
| Ki-67 | Alexa Fluor 700 | SolA15 | eBioscience |
| KLRG1 | APC | 2F1 | BD Biosciences |
| KLRG1 | PE-CF594 | 2F1 | BD Biosciences |
| MHCII | Alexa Fluor 700 | M5/114.15.3 | eBioscience |
| MHCII | Brilliant Violet 650 | M5/114.15.2 | BD Biosciences |
| NK1.1 | PE | PK136 | eBiosciences |
| NK1.1 | APC | PK136 | BD Biosciences |
| RORγt | PE | Q31-378 | BD Biosciences |
| Sca-1 | Pacific Blue | D7 | BioLegend |
| Sca-1 | Brilliant Violet 786 | D7 | BD Biosciences |
| Siglec-F | PE | E50-2440 | BD Biosciences |
| Siglec-F | PE-CF594 | E50-2440 | BD Biosciences |
| Streptavidin | APC-eFluor 780 |  | eBioscience |
| Streptavidin | Brilliant Violet 786 |  | BD Biosciences |
| T1/ST2 | Biotin | DJ8 | MD Bioproducts |
| T1/ST2 | FITC | DJ8 | MD Bioproducts |
| T-bet | Brilliant Violet 421 | O4-46 | BD Biosciences |
| TER-119 | PE | TER-119 | eBioscience |

Table S2 Antibodies used for confocal microscopy.

| **Antibody** | **Conjugate** | **Clone** | **Company** |
| --- | --- | --- | --- |
| B220 | Biotin | TY25 | eBioscience |
| CD3 | Biotin | 145-2C11 | BD Biosciences |
| αrat | Cy5 | Polyclonal | Jackson ImmunoResearch |
| αhamster | Cy3 | Polyclonal | Jackson ImmunoResearch |
