# Supplementary material for: T cells and ILC2s are major effector cells in influenza‐induced exacerbation of allergic airway inflammation in mice
Source: Eur J Immunol. 2018 Jun 11;49(1):144–56. doi: 10.1002/eji.201747421 (PMC6585726; doi:10.1002/eji.201747421)
Supplement: Supplementary file 1 — Peer review correspondence [file EJI-49-144-s001.pdf]

European Journal of Immunology

T cells and ILC2s are major effector cells in influenza-induced exacerbation of allergic airway inflammation in mouse

Bobby W.S. Li, Marjolein J.W. de Bruijn, Melanie Lukkes, Menno van Nimwegen, Ingrid M. Bergen, Alex KleinJan, Corine H. GeurtsvanKessel, Arno Andeweg, Guus F. Rimmelzwaan and Rudi W. Hendriks

Correspondence: Dr. Rudi W. Hendriks, Department of Pulmonary Medicine Erasmus MC Rotterdam , 3000 CA Rotterdam, The Netherlands

---

Review Timeline:	Submission date:	17-Nov-2017
	First Editorial decision:	21-Dec-2017
	Revision received:	14-Mar-2018
	Accepted:	09-May-2018

Handling Executive Committee member: Prof. Annette Oxenius

Please note that the correspondence below does not include the standard editorial instructions regarding preparation and submission of revised manuscripts, only the scientific revisions requested and addressed.

First Editorial Decision
21-Dec-2017

Dear Mr. Li,

Manuscript ID eji.201747421 entitled "T cells and ILC2s are major effector cells in influenza-induced asthma exacerbation" which you submitted to the European Journal of Immunology has been reviewed. The comments of the referees are included at the bottom of this letter.

A revised version of your manuscript that takes into account the comments of the referees will be

reconsidered for publication. Should you disagree with any of the referees ' concerns, you should address this in your point-by-point response and provide solid scientific reasons for why you will not make the requested changes.

You should also pay close attention to the editorial comments included below. **In particular, please edit your figure legends to follow Journal standards as outlined in the editorial comments. Please state the number of independent experiments and the number of samples per experiment. For flow cytometry results, please also show the full gaing strategy. Failure to do this will result in delays in the re-review process.**

Please note that submitting a revision of your manuscript does not guarantee eventual acceptance, and that your revision will be re-reviewed by the referees before a decision is rendered.

If the revision of the paper is expected to take more than three months, please inform the editorial office. Revisions taking longer than six months may be assessed by new referee(s) to ensure the relevance and timeliness of the data.

Once again, thank you for submitting your manuscript to European Journal of Immunology and we look forward to receiving your revision.

Yours sincerely,
Marta Vuerich

On behalf of
Prof. Annette Oxenius

Dr. Marta Vuerich
Editorial Office
European Journal of Immunology
e-mail: ejied@wiley.com
www.eji-journal.eu

****

Reviewer: 1

Comments to the Author

Influenza virus infection is an important cause of severe asthma exacerbations, but it remains unclear how a Th1-mediated antiviral response triggers a prototypical Th2 disease. In this manuscript Dr. Hendriks and colleagues investigated CD4+ T cells and group 2 innate lymphoid cells (ILC2s) in influenza virus-infected mice and found that ILC2s accumulated in the lung rapidly after influenza virus infection, but the induction of IL-5 and IL-13 secretion was delayed and concomitant with T cell activation. In an influenza-induced asthma exacerbation model the authors further noticed an initial reduction of ILC2 numbers and cytokine production in the broncho-alveolar lavage compared to chronic house dust mite (HDM)-mediated airway inflammation alone. ILC2s had a phenotype characterized by low T1/ST2, ICOS, KLRG1 and CD25 expression, resembling naive ILC2s. Consequently, the contribution of ILC2s to type 2 cytokine production in the early stage of the influenza-induced asthma exacerbation was limited. In contrast, T cells showed increased IL-4 and IL-5 production when exposed to both HDM and influenza virus. Nevertheless, upon clearance of the virus, ILC2s regained an activated T1/ST2highICOShighKLRG1highCD25high phenotype paired with cytokine production and were major contributors to the type 2 cytokine milieu. Collectively, the observations by the authors nicely demonstrate that both T cells and ILC2s contribute to influenza-induced asthma exacerbation, but with different kinetics.
This is beautiful manuscript. Rationale and hypothesis clearly displayed, experiments well chosen and performed, claims are substantiated by the data. The work details important findings on how pulmonary viral infections contribute to pulmonary allergic reactions and clarifies some uncertainties and controversies regarding the involvement of ILC2. I only have minor comments that should be easy to address to strengthen the clarity and message of the paper.

ïƒ Figure 2B: Regarding the identification of ILC2 it would be important to extend the FACS gating and add/show expression for ST2, ICOS, Thy1, KLRG1.

ïƒ Figure 2E & S1: Are Gata3+ cells really ILC2? Gata3 is not a determining factor of ILC2 only as many cells in the lungs (including other ILCs, Th2, Treg cells). As such the data should be interpreted with more caution.

ïƒ Intracellular cytokine staining need to be shown with controls throughout the manuscript e.g. Figure 3B, Figure 5; isotype and FMO controls need to be shown for IL-5 and IL-13.

ïƒ The choice of the Influenza strain should be justified and discussed in light of other data published on this topic (Artis, Umetsu, Fritz, Metzger) â€“ how strain specific are the observations?

ïƒ Gating strategies for eosinophils, neutrophils, B and T cells together with ILC2 should be shown clearly (FACS plots gating and explained in the methods section with antibody clones).

European Journal of Immunology

ïƒ Were other cells implicated in type 2 immune responses such as type 2 DCs, basophils, mast cells, M2 macrophages analyzed? If the authors have data on it I would like to invite them to add it.

ïƒ Figure 4E: scale bars should be added to the pictures.

ïƒ All flow cytometry antibody clones and sources should be given in the methods sections. ILC2 staining and intracellular staining procedure for cytokine analysis should be detailed clearly. Also the tissue digestion protocol and live-dead/duplex exclusion should be written in more details.

Reviewer: 2

Comments to the Author
The authors characterize the contribution of ILC2 and CD4 T cells to type 2 immunity following infection with influenza and in a model of influenza-induced (HDM) asthma exacerbation.

The paper is largely descriptive and does not provide much mechanistic insight. On the other hand, the experiments are performed to a high standard and provide a good and thorough overview of type 2 immune cells in the two models.

The paper seems publishable in the Journal with one caveat. It is entirely unclear from the data if the infection of mice HDM pre-treated with influenza leads to asthma exacerbation (Figure 4). Airway resistance should be evaluated in the various treatment groups and correlated with type 2 immunity. Without these data the major claim of the paper is not supported by the data.

First Revision – authors' response

14_Mar-2018

Point-by-point reply to:

T cells and ILC2s are major effector cells in influenza-induced exacerbation of allergic airway inflammation

Bobby W.S. Li[1], Marjolein J.W. de Bruijn[1], Melanie Lukkes[1], Menno van Nimwegen[1], Ingrid M. Bergen[1], Corine H. GeurtsvanKessel[2], A. KleinJan[1], Arno Andeweg[2], Guus F. Rimmelzwaan[2] and Rudi W. Hendriks[1]
[1]Department of Pulmonary Medicine, Erasmus MC Rotterdam, Rotterdam, the Netherlands
[2]Department of Viroscience, Erasmus MC Rotterdam, Rotterdam, the Netherlands

Reviewer 1

Influenza virus infection is an important cause of severe asthma exacerbations, but it remains unclear how a Th1-mediated antiviral response triggers a prototypical Th2 disease. In this manuscript Dr. Hendriks and colleagues investigated $CD4^{+}$ T cells and group 2 innate lymphoid cells (ILC2s) in influenza virus-infected mice and found that ILC2s accumulated in the lung rapidly after influenza virus infection, but the induction of IL-5 and IL-13 secretion was delayed and concomitant with T cell activation. In an influenza-induced asthma exacerbation model the authors further noticed an initial reduction of ILC2 numbers and cytokine production in the broncho-alveolar lavage compared to chronic house dust mite (HDM)-mediated airway inflammation alone. ILC2s had a phenotype characterized by low T1/ST2, ICOS, KLRG1 and CD25 expression, resembling naïve ILC2s. Consequently, the contribution of ILC2s to type 2 cytokine production in the early stage of the influenza-induced asthma exacerbation was limited. In contrast, T cells showed increased IL-4 and IL-5 production when exposed to both HDM and influenza virus. Nevertheless, upon clearance of the virus, ILC2s regained an activated $T1/ST2^{high}ICOS^{high}KLRG1^{high}CD25^{high}$ phenotype paired with cytokine production and were major contributors to the type 2 cytokine milieu. Collectively, the observations by the authors nicely demonstrate that both T cells and ILC2s contribute to influenza-induced asthma exacerbation, but with different kinetics.
This is beautiful manuscript. Rationale and hypothesis clearly displayed, experiments well-chosen and performed, claims are substantiated by the data. The work details important findings on how pulmonary viral infections contribute to pulmonary allergic reactions and clarifies some uncertainties and controversies regarding the involvement of ILC2. I only have minor comments that should be easy to address to strengthen the clarity and message of the paper.
- Reply: We thank the referee for these positive remarks.

Comments

1. Figure 2B: Regarding the identification of ILC2 it would be important to extend the FACS gating and add/show expression for ST2, ICOS, Thy1, KLRG1.
- Reply: We have extended the FACS gating and now show surface expression of ICOS, KLRG1 and T1/ST2 on the gated ILC2 in Figure 2B. Figure legends are adapted accordingly. We have previously shown that ILC2 gated in this way consistently express high levels of Thy1 following IL-33 or HDM exposure (Li et al. Front Immunol. 2017).

2. Figure 2E & S1: Are Gata3+ cells really ILC2? Gata3 is not a determining factor of ILC2 only as many cells in the lungs (including other ILCs, Th2, Treg cells). As such the data should be interpreted with more caution.
- Reply: We agree that other cell types are known to express GATA3, including ILC1 and IL3, CD4 and CD8 cells. However, in our hands, confocal microscopy analysis only detects cells that express high levels of GATA3. We detect YFP only in a small fraction of T cells (Th2 cells) and not in all CD4+ and CD8+ T cells (which do express GATA3, but at low levels). We have now added a sentence to the legends of Figure 2 and Figure S1 to provide additional clarification.

3. Intracellular cytokine staining need to be shown with controls throughout the manuscript e.g. Figure 3B, Figure 5; isotype and FMO controls need to be shown for IL-5 and IL-13.

- Reply: As a standard procedure, we use gated cells of which we know that they are negative for the cytokine as a control, rather than isotypes or FMO. The point is here that controls such as isotypes or FMOs would not detect non-specific binding of the antibody. In the case of Figure 3B, we have placed the quadrant on the basis of cells of which we know are IL-5 and IL-13 negative (Lineage^{+}CD4^{-}).

4. The choice of the Influenza strain should be justified and discussed in light of other data published on this topic (Artis, Umetsu, Fritz, Metzger) – how strain specific are the observations?
- Reply: We thank the reviewer for this insight and have included an extra paragraph in the discussion regarding low- and high-pathological influenza virus strains (page 14, lines 21 – 25 and page 15, lines 1 – 2). Importantly, a marked innate response followed by an accumulation of eosinophils, neutrophils, dendritic cells and T cells was previously reported in an influenza virus infection model preceded by chronic exposure to HDM (Ravanetti et al. Allergy, 2016). In these experiments X31 was used, but ILC2s were not studied. We have now mention this in the introduction (page 5, line 14).

5. Gating strategies for eosinophils, neutrophils, B and T cells together with ILC2 should be shown clearly (FACS plots gating and explained in the methods section with antibody clones).
- Reply: We have added the FACS gating strategy of eosinophils, neutrophils, B cells, CD4+ T cells and CD8+ T cells as Supplementary Figure S3.

6. Were other cells implicated in type 2 immune responses such as type 2 DCs, basophils, mast cells, M2 macrophages analyzed? If the authors have data on it I would like to invite them to add it.
- Reply: We thank the reviewer for this suggestion. We have added histology data on mast cells in Figure 4F and have adapted the methods and results section accordingly. In addition, we have elaborated on the findings in the context of mast cell – ILC2 interaction in the discussion (page 16 lines 7 – 14).

7. Figure 4E: scale bars should be added to the pictures.
- Reply: Image scales were not recorded. However we have added the magnification factor in the figure legends.

8. All flow cytometry antibody clones and sources should be given in the methods sections. ILC2 staining and intracellular staining procedure for cytokine analysis should be detailed clearly. Also the tissue digestion protocol and live-dead/duplex exclusion should be written in more details.
- Reply: We now provide a list of antibodies (including clones and suppliers) used for flow cytometry and confocal microscopy in the supplementary data (Supplementary Tables S1 and S2). We have further clarified the tissue preparation and staining protocols as well as the gating strategy in the methods section (page 17, lines 10 – 20).

Reviewer 2
The authors characterize the contribution of ILC2 and CD4 T cells to type 2 immunity following infection with influenza and in a model of influenza-induced (HDM) asthma exacerbation.
The paper is largely descriptive and does not provide much mechanistic insight. On the other hand, the experiments are performed to a high standard and provide a good and thorough overview of type 2 immune cells in the two models.
The paper seems publishable in the Journal with one caveat. It is entirely unclear from the data if the infection of mice HDM pre-treated with influenza leads to asthma exacerbation (Figure 4). Airway resistance should be evaluated in the various treatment groups and correlated with type 2 immunity. Without these data the major claim of the paper is not supported by the data.
- Reply: We agree with reviewer 2 that airway resistance is an important parameter in the quantitative evaluation of asthma exacerbations. However, in our experience in the house dust mite models, lung function measurements are not highly sensitive. Therefore, we expect that detection of differences between chronic HDM alone and chronic HDM + influenza virus infection will require large numbers of mice. Moreover, given the complex kinetics in the contribution of T cells and IlC2, such an analysis should be done at several time points. A meaningful analysis of airway resistance would therefore require so many mice, that our ethical committee likely would not allow these experiments. Most importantly, these experiments would not address our key question which cells are responsible for the exacerbation of the type II response, T cells or ILC2? Nevertheless, we do agree with the referee that an increase in inflammation would not necessarily result in increased airway resistance. We have therefore changed the wording throughout the manuscript and replaced "asthma exacerbation" with "exacerbation of allergic airway inflammation".

Second Editorial Decision

09-Apr-2018

Dear Dr. Hendriks,

It is a pleasure to provisionally accept your manuscript entitled "T cells and ILC2s are major effector cells in influenza-induced exacerbation of allergic airway inflammation" for publication in the European Journal of Immunology. For final acceptance, please follow the instructions below and return the requested items as soon as possible as we cannot process your manuscript further until all items listed below are dealt with.

Please note that EJI articles are now published online a few days after final acceptance (see Accepted Articles: https://onlinelibrary.wiley.com/toc/15214141/0/ja). The files used for the Accepted Articles are the final files and information supplied by you in Manuscript Central. You should therefore check that all the information (including author names) is correct as changes will NOT be permitted until the proofs stage.

We look forward to hearing from you and thank you for submitting your manuscript to the European Journal of Immunology.

Yours sincerely,
Marta Vuerich

on behalf of
Prof. Annette Oxenius

Dr. Marta Vuerich
Editorial Office
European Journal of Immunology
e-mail: ejied@wiley.com
www.eji-journal.eu
